# Silent Atrial Fibrillation: Definition, Clarification, and Unanswered Issues

**DOI:** 10.1111/anec.12307

**Published:** 2015-10-08

**Authors:** Harold L. Kennedy

**Affiliations:** ^1^ Cardiovascular Disease and Medicine University of South Florida Tampa FL

**Keywords:** silent atrial fibrillation, atrial fibrillation burden, subclinical atrial fibrillation

## Abstract

Silent or subclinical asymptomatic atrial fibrillation has currently gained wide interest in the epidemiologic, neurologic and cardiovascular communities. The association of brief episodes of paroxysmal atrial fibrillation or surrogate atrial arrhythmias which predict future clinical adverse events have been established. Nevertheless there exists a confounding array of definitions to indicate its presence without discrete indication of which populations should be examined. Moreover the term “atrial fibrillation burden” (AFB) has emerged from such studies with a plethora of descriptions to prognosticate both arrhythmic and clinical adverse events. This presentation suggests clarification of diagnostic definitions associated with silent atrial fibrillation, and a more precise description of AFB. It examines the populations across the current disease and cardiovascular invasive therapeutic spectrum that lead to both silent atrial fibrillation and AFB. It describes the diagnostic methods of arrhythmia detection utilizing the surface ECG, subcutaneous ECG or intra‐cardiac devices and their relationship in seeking meaningful arrhythmic markers of silent atrial fibrillation. Whereas a wide range of *clinical risk factors* of silent atrial fibrillation have been validated in the literature, there is an ongoing search for those *arrhythmic risk factors* that precisely identify and prognosticate outcome events in diverse populations at risk of atrial fibrillation and its complications. This presentation identifies this chaos, and focuses attention on the issues to be addressed to facilitate descriptive and comparative scientific studies in the future. It is a call to action specifically to the medical arrhythmic community and its specialty societies (i.e., ISHNE, HRS, EHRA) to begin a quest to unravel the arrhythmic quagmire associated with “silent atrial fibrillation.”

## Introduction

The importance of clinical atrial fibrillation to all physicians has emerged during the last decade.^1^, ^2^ Data of 2010 taken from the Global Burden of Disease study has shown an estimated prevalence of 33.5 million persons affecting 2.5–3.2% (mean 3%) of populations across all countries on many continents.^2^ The addition of 5 million new cases annually currently, and the data from 1990 indicates that the incidence has steadily increased over the past two decades.^2^ Whereas aging of the population with its inverse pyramid demographics of the post‐WWII era has been a factor in secular trends, improvements in survival resulting from improved medical therapies have also contributed to the enlarging elderly population. Developed countries, especially those of North America, were found to have higher prevalence rates of atrial fibrillation, and it was most pronounced in men than in women.^2^ The lowest prevalence rates globally were found for both men and women in the Asia–Pacific area.^2^

Clinically in North America an appreciation of the precipitants of atrial fibrillation was recently reported to account for one‐third of all occurrences of atrial fibrillation, and resulted from surgery, infection, and myocardial infarction.^3^ Of course to the clinical physician the complications of atrial fibrillation which include emboli, heart failure and early mortality are the most important consequences of the arrhythmia. Cerebral emboli leading to ischemic stroke and cognitive decline are the most emotionally dreaded events by both patient and physician,^4^ and account for 25–30% of all acute ischemic strokes.^2^ Atrial fibrillation increases stroke risk fivefold, and has been demonstrated to increase both cardiovascular and all‐cause mortality.^4^, ^5^ In the United States stroke is the fifth leading cause of death, and a leading cause of chronic severe disability.^6^ Thus, it is timely for physicians to focus their attention and efforts on the detection, prevention and therapy of atrial fibrillation.^7^, ^8^

Although a variety of clinical risk markers have emerged to identify the patient at risk of atrial fibrillation during the past 3 decades,^9^, ^10^ recent guidelines most commonly have recommended the CHA_2_DS_2_‐VASc score to detect such susceptible persons.^8^, ^11^ Accordingly, arrhythmia specialist physicians (both noninvasive and invasive) are interested and seeking the detection and therapy of atrial fibrillation in a variety of populations. These efforts have emerged according to the interests, knowledge and resources of a variety of stakeholders within both academia and industry, and while contributing new data and information … have also resulted in a plethora of unclear definitions without standardization of terminology or reported parameters. This presentation seeks to examine this quandary, provide some definitions, and present unanswered issues that need to be addressed by the scientific community at large. The need for such a focus and clarification has been appreciated by many authors recently, 12, 13, 14, 15, 16 and should prove valuable for future research of atrial fibrillation.

## Definitions

**Figure 1:**
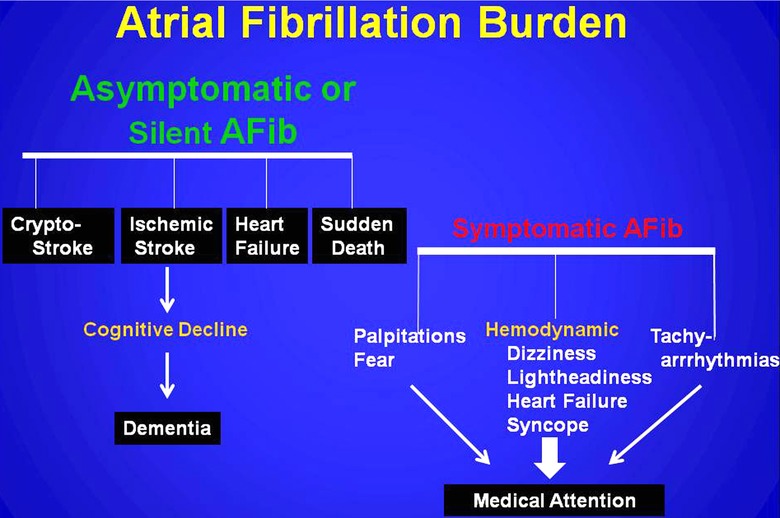
The clinical presentations of symptomatic atrial fibrillation (AFib) or asymptomatic atrial fibrillation.

It is not surprising that arrhythmia specialist physicians have also sought to identify arrhythmic risk markers that predict adverse atrial fibrillation events to complement the clinical risk marker of CHA_2_DS_2_‐VASc. From such efforts have emerged the term *"silent atrial fibrillation"* (SAFib) which initially commutated the occurrence and detection of subclinical asymptomatic episodes of paroxysmal atrial fibrillation. To quantitate such episodes of asymptomatic SAFib there emerged the concept of "*atrial fibrillation burden"* (AFB). AFB has been represented by various arrhythmic markers, predominantly supraventricular arrhythmias, which prognosticate the development of atrial fibrillation and/or its outcomes. Whereas patients with symptomatic atrial fibrillation are usually discovered by medical attention resulting from symptoms associated with hemodynamic complaints, unfortunately SAFib may only present after the most serious of complications such as ischemic stroke or sudden death (Fig. 1). The term AFB has also evolved to represent a variety of supraventricular arrhythmic markers (see below) which prognosticate either the development of paroxysmal or persistent atrial fibrillation and/or its adverse outcomes. Perhaps the terms *AFB‐arrhythmic* and *AFB‐outcomes* should be promulgated and standardized to identify these separate burdens more clearly to physicians at large.

## Populations

**Figure 2:**
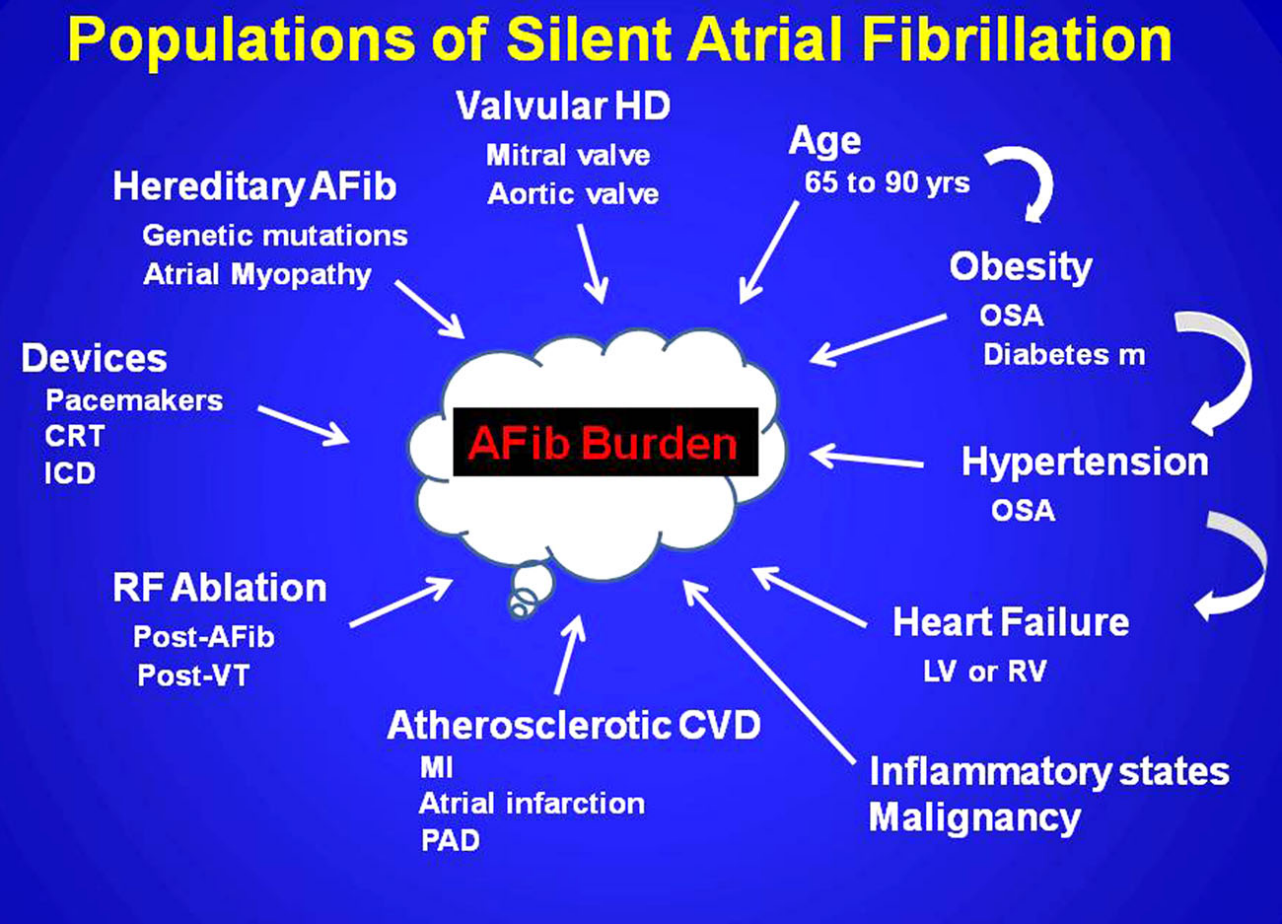
Populations described with silent atrial fibrillation and their influence on atrial fibrillation burden. AFib = atrial fibrillation.

The populations of SAFib and AFB are diverse and have contributed to the confounding of clear interpretation of the data (Fig. 2). The myriad of mechanisms which lead to atrial changes through atrial myopathy mechanisms, inflammation, direct invasion, or volume overload and their consequence occur across the current disease and invasive therapeutic spectrum (Fig. 2).^2^, ^17^ Thus there has been a plethora of scientific reports emanating from a variety of medical stakeholders who predominantly are composed of the epidemiology, neurology, and cardiovascular community. Thus a need for standardized communication within these disciplines would seem to be useful in future research endeavors.

### Arrhythmia Detection

**Figure 3:**
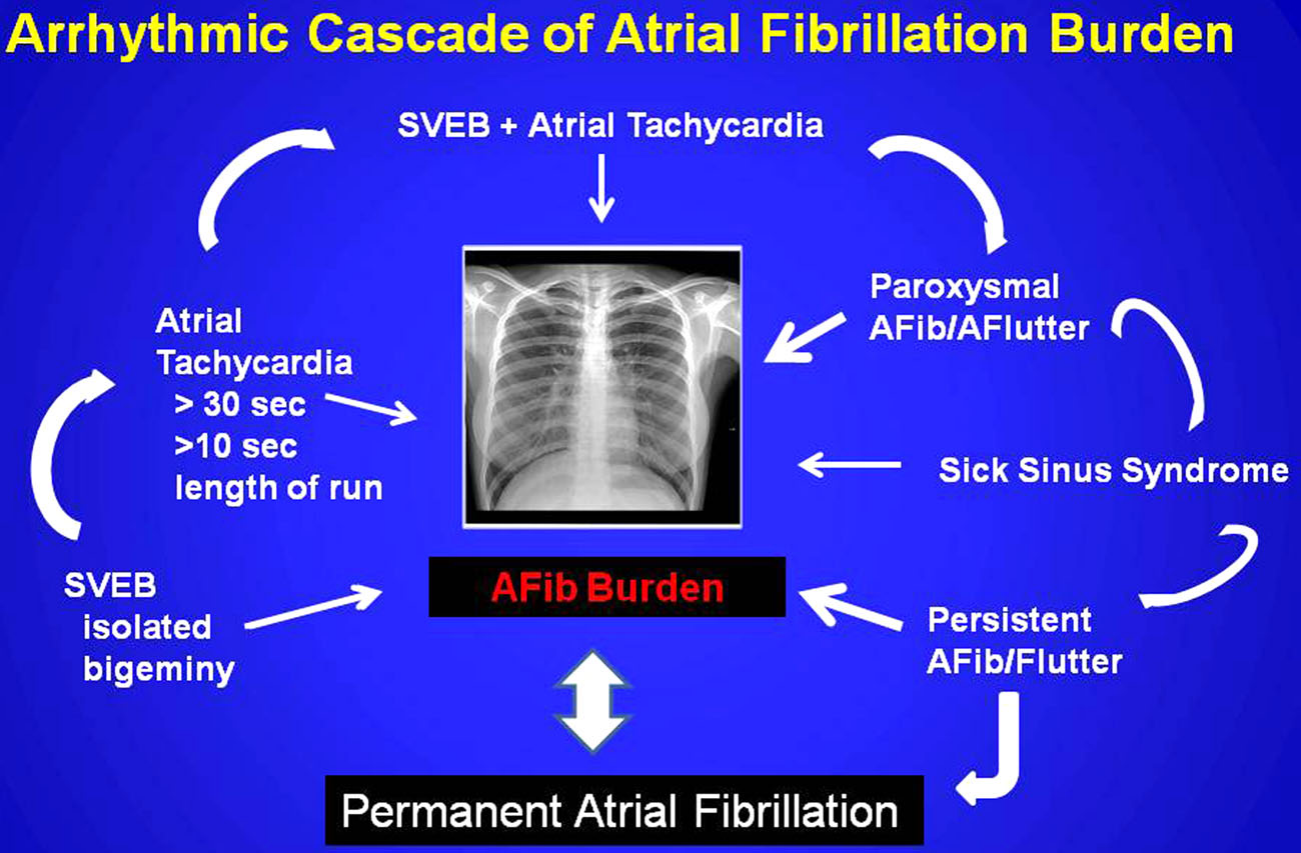
Described arrhythmic markers contributing to the cascade of Atrial Fibrillation Burden resulting in permanent atrial fibrillation. AFib = atrial fibrillation.

**Figure 4:**
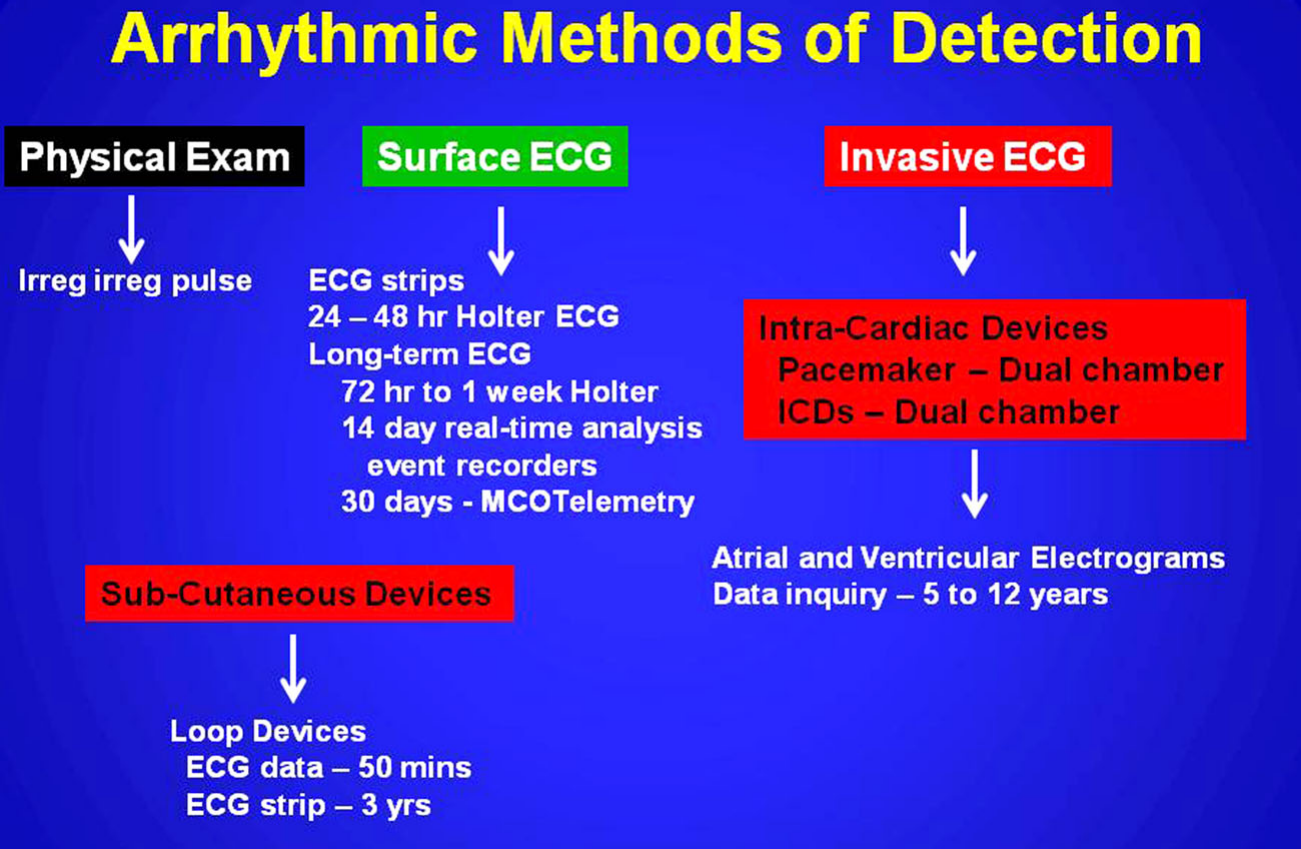
Arrhythmic methods of detection currently utilized to detect atrial fibrillation.

SAFib detection currently seems most directed at a wide variety of arrhythmic markers which collectively or independently can identify a critical AFB or the occurrence of persistent or permanent atrial fibrillation (Fig. 3). This detection employs a host of approaches which include physical examination, surface electrocardiographic (ECG) recordings, or invasive electrocardiographic devices (Fig. 4).^18^, ^19^ Accordingly such methods have advanced our understanding of SAFib, but also have confounded and constricted our vision of the detection that emanates from the methodology employed. Whereas a lack of fiscal resources secondary to the current global financial crisis has affected the academic community at large, industry has been resourceful in offering a variety of approaches with emerging digital resources within invasive devices to detect SAFib‐A burden. This invasive approach, however, in many countries would impose a heavy unsustainable financial burden on their health care system, but nonetheless in developed nations the cost currently seems justified without a proven alternative noninvasive algorithm of investigation. Therefore efforts to standardize such research, establish validated algorithms of investigation in these diverse populations, and assess standardized arrhythmic markers for their prognostic outcomes is badly needed. 12, 13, 14, 15, 16

A noncomplete review of arrhythmic markers appearing in the recent medical literature from diverse detection methods on a variety of medical instruments in multiple populations, illustrates and highlights the current existing chaos (Table 1). 20, 21, 22, 23, 24, 25, 26, 27, 28, 29, 30, 31, 32, 33, 34, 35, 36, 37 Moreover, there is an appreciation that current clinical trials in progress predominantly employ relatively expensive invasive devices that advance our understanding without recognition of perhaps less costly noninvasive methodologies that could be employed if an investigative guideline algorithm existed. This is not to denigrate industry which must serve its commercial aspirations for success, but to focus thought in the academic community upon its responsibility to define cost‐effective validated methods and investigative algorithms which render quality outcomes for society in general.^38^, ^39^ Such algorithms and guidelines would enhance current clinical trials and studies, health care systems, insurance carriers and government reimbursement policies.^38^, ^39^

**Table 1:** Literature Reports of Criteria and Populations Described to Have Silent Atrial Fibrillation

Detection Method	Diagnostic Criteria	Population	Publication
**Surface ECG Studies**			
1. 24 Hr Holter	> 30 PAB/hr	Community ‐ CVHS	Dewland TA (20)
2. 24 Hr Holter	>70 PAB/hr	Acute Ischemic Stroke	Wallmann D (21)
3. 24 Hr Holter	>218 PAB/hr	Men Born in 1914	Engstrom G (22)
		Community	
4. 24 Hr Holter	SVT >3 beats < 30 sec	Ischemic Strokes	Arsava EM (23)
	AF > 30 sec		
5. 48 Hr Holter	> 30 PAB/hr	Copenhagen Population	Binici Z (24)
	SVT > 20 beats		
6. 72 Hr Holter	AF > 30 sec	Ischemic Stroke	Grond M (25)
7. 7 day Holter	AFB	Post ‐ RF Ablation	Winkle RA (26)
8. 7 day Holter	AF >30 sec	Post ‐ RF Ablation	Gang UFO (27)
	>142 PAB/day		
9. 14 day Real‐time analysis	AFB	AF patients	Rosenberg MA (28)
10. 28 day ‐ 2x/day 30 sec	AF > 30 sec	Palpitations/Dizziness	Hendrikx T (29)
Transtelephonic ECG	SVT > 30 sec	Lightheadiness	
11. 30 day ‐ 2x/day 10 sec	Irreg irreg > 10 sec	Symptomatic Paroxysmal AF	Doliwa PS (30)
Transtelephonic ECG			
12. 30 day loop event recorder	> 100 PAB/day	Cryptogenic Stroke	Gladstone DJ (31)
	Irreg RR > 30 beats	TIA	
	Irreg RR > 30 sec		
13. 28 days MCOT	Irreg irreg > 10 sec	Symptomatic Paroxysmal AF	Favilla CG (32)
14. iPhone	RMSSD + Shannon Entropy	AF Cardioversion	McManus DD (33)
**Sub‐cutaneous ECG**			
15. 1 day to 3 years	Irreg irreg > 30 sec algorithm	Stroke or TIA < 90 days	Sanna T (34)
Sub‐cutateous ECG			
**Intra‐Cardiac ECG**			
16. 6 years duration	SVT atrial rate > 220 bpm > 5 min	DDDR vs VVIR pacing	Glotzer TV (35)
Pacemaker			
17. 3 mo duration	Atrial rate > 190 bpm	Hypertension population with SSS/AV node disease	Healey JS (36)
Pacemaker + ICD	> 6 min		
18. 14 mo duration	Atrial tachy/AFB	Pacemaker, ICD, CRT	Ziegler PD (37)
Pacemaker + ICD + CRT	> 6 hrs/day	Population	

PAB = premature atrial beats; CVHS = Cardiovascular Health Study; SVT = supraventricular tachycardia; AF = atrial fibrillation; AFB = atrial fibrillation burden; RF = radio frequency; TIA = transient ischemic attack; RMSSD = root mean square succesive differences; ICD = implantable cardiac defibrillator; CRT = cardiac resynchronization therapy.

### Risk Factors of SAFib‐A and SAFib‐O Burden

*Clinical risk factors* that identify the population most likely to demonstrate a significant arrhythmic marker or SAFib‐A burden are shown in Table 2. These clinical risk factors have a robust association to the various arrhythmic definitions defined in the current medical literature (Table 2). Curiously, patients of blood group O seem to enjoy a reduced susceptibility to developing atrial fibrillation and its outcomes.^40^

**Table 2:** Clinical Risk Factors to Detect Silent Atrial Fibrillation

Age >75 years
Cryptogenic stroke
Ischemic stroke
Neurological disease
Hypertensive heart disease
Diabetes mellitus
Obesity
Obstructive sleep apnea
RF ablation
ICD or pacemaker
Post‐afib precipitant
Mitral valve disease
High CHA_2_DS_2_‐VASc score

*Arrhythmic risk factors* that precisely identify and prognosticate outcome events in populations at risk are the "holy grail quest" of current arrhythmia investigations. The difficulty of this in the current confounded milieu is evident and noted by others.^41^ As shown in Table 3, the clinical age of the patient, patient population, and CHA_2_DS_2_‐VASc score undeniably have been established to influence the SAFib‐O burden and outcome. But the definitive criteria or investigative algorithm of employing which diagnostic devices within a specific population for what specific durations with defined criteria of SAFib‐A or SAFib‐O burdens do not always exist. Furthermore the relative ease of anticoagulation specific to avert many of the complications of atrial fibrillation provided by the new class of antithrombin or Factor X oral anticoagulants with their enhanced benefit on outcomes and safety make this a scientific question of some urgency. Even the time of examination in various populations, and an appreciation of the lack of temporal relationship between the examination and outcome event … further confounds the complexity of investigation.^42^ The investigative community must nevertheless take up and engage this clinical challenge.

**Table 3:** Clinical Risk Factors Affecting AFB and Outcomes

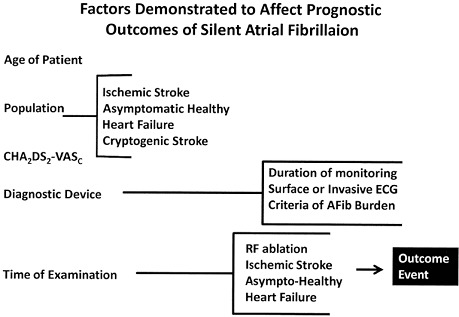

### Issues to Be Addressed

Several issues come to the fore in addressing the clinical challenge of SAFib and include the following:

1. *An authoritative consensus of populations at greatest risk*. Whereas all persons >75 years of age, stroke patients (cryptogenic and ischemic), intracardiac device patients, post‐RF ablation, and high CHA_2_DS_2_‐VASc score patients seemingly are clearly candidates to be identified in such a consensus document. What about the populations of obesity, sleep apnea, convulsive disorders, syncope, malignancy,post‐precipitant atrial fibrillation and inflammatory conditions? What diagnostic algorithm or criteria should exist for low risk populations?
2. *A diagnostic algorithm of examination in a specific population*. Although some populations can employ the most simple of diagnostic methods (i.e., palpation of pulse), should there be a gradient guideline of cost‐effective diagnostic testing utilizing recommended surface ECG techniques (e.g., ECG rythmn strip, standard ECG, etc.) before employing costly invasive devices in specific populations?
3. *When to examine in each population at risk*.
  An authoritative consensus could guide a standardized time of examination and its duration for meaningful detection of SAFib‐A and SAFib‐O. This would better guide the scientific community in its requisition of tests and applications of therapies. It would render cost‐efficiencies to ongoing clinical studies and randomized trials.
4. *Establishment of Definitive Criteria for SAFib‐A and SAFib‐O*
  By establishing definitive criteria of SAFib‐A in specific populations a more robust scientific literature and knowledge base would be established. Perhaps a time‐threshold effect could be defined, whereby a greater burden of atrial fibrillation or longer episodes of atrial fibrillation should confer a greater risk of adverse outcomes. Prevention and therapies would be better guided and adverse outcomes hopefully avoided. The knowledge base of those SAFib‐O markers would be better appreciated by the medical community at large, and the overall medical community would be guided to lower the global burden of atrial fibrillation and is complications.

## Summary

This presentation is a call to action specifically to the medical arrhythmic community and its specific specialty societies (i.e., ISHNE, HRS, EHRA) to begin an effort to address the quagmire of SAFib. This dilemma currently exists, and should not be left to commercial market forces alone, but should receive thoughtful, incisive and cost‐effective attention from physicians for the benefit of their patients and society at large.
